# Longitudinal wall fractional shortening: an M-mode index based on mitral annular plane systolic excursion (MAPSE) that correlates and predicts left ventricular longitudinal strain (LVLS) in intensive care patients

**DOI:** 10.1186/s13054-017-1876-x

**Published:** 2017-11-25

**Authors:** Stephen J. Huang, Iris Ting, Andrea M. Huang, Michel Slama, Anthony S. McLean

**Affiliations:** 1 Department of Intensive Care Medicine, Nepean Hospital, University of Sydney, Sydney, NSW 2747 Australia; 2 Cardiovascular Ultrasound Laboratory, Nepean Hospital, Sydney, NSW Australia; 3 Sydney Medical Program, Nepean Clinical School, University of Sydney, Sydney, NSW Australia; 4 Unité de réanimation médicale CHU Sud Amiens, and unité INSERM 1088, UPJV, Amiens, France

**Keywords:** Left ventricular function, MAPSE, M-mode, Longitudinal strain, Speckle tracking

## Abstract

**Background:**

Left ventricular longitudinal strain (LVLS) is a modern measurement for LV function. However, strain measurement is often difficult in critically ill patients. We sought to show LVLS can be estimated using M-mode-derived longitudinal wall fractional shortening (LWFS), which is less dependent on image quality and is easier to perform in critically ill patients.

**Methods:**

Transthoracic echocardiographic records were retrospectively screened and 80 studies suitable for strain and M-mode measurements in the apical 4-chamber view were selected. Longitudinal wall fractional shortening was derived from conventional M-mode (LWFS) and curved anatomical M-mode (CAMMFS). The relationships between LVLS and mitral annular plane systolic excusion (MAPSE) and M-mode-derived fractional shortening were examined using univariate generalized linear model in a training set (*n* = 50) and was validated in a separate validation set (*n* = 30).

**Results:**

MAPSE, CAMMFS, and LWFS demonstrated very good correlations with LVLS (*r* = 0.852, 0.875 and 0.909, respectively). LWFS was the best unbiased predictor for LVLS (LVLS = 1.180 x LWFS - 0.737, *P* < 0.001). Intra- and inter-rater agreement and reliability for LWFS measurement were good.

**Conclusions:**

LVLS can be estimated by LWFS in the critically ill patients. It provides a fast and accurate prediction of LVLS. LWFS is a reproducible and reliable measurement which can be used as a potential index in place of LVLS in the critically ill population.

**Electronic supplementary material:**

The online version of this article (doi:10.1186/s13054-017-1876-x) contains supplementary material, which is available to authorized users.

## Background

Global longitudinal strain (GLS) is a modern clinical utility that has superior sensitivity in detecting early cardiac dysfunction before clinical manifestations [1, 2]. For example, strain was able to identify impaired ventricular function in patients with early septic shock but preserved ejection fractions [3]. GLS can also be used for predicting outcomes in patients with heart failure and myocardial infarction [4, 5]. However, transthoracic echocardiographic (TTE) images are often suboptimal for strain measurement in the critically ill patients. To be a useful left ventricular (LV) systolic function marker in the critical care setting, the marker should be easily obtained even if the image quality is suboptimal.

Myocardial strain is most commonly defined as “deformation of the myocardium” and is usually measured by speckle-tracking echocardiography in modern machines [6, 7]. To many critical care physicians, the meaning of “strain” (a negative number) and “deformation” is not as intuitive as other traditional indices such as ejection fraction and dP/dt. Further, different definitions of GLS are adopted by different researchers and vendors, adding further complexity to interpretations and usage. For example, some define GLS as the change in length for the “entire U-shaped length of LV”, whilst others define GLS as the average of the 17 segments [8, 9]. Although mathematical definition of longitudinal strain is often quoted as “change in length divided by its original length” in the literature, the fact that the definition is simply an expression of fractional shortening is less readily appreciated.

Mitral annular plane systolic excursion (MAPSE) is a reliable marker for LV systolic function and is less dependent on image quality [10]. We proposed that LV longitudinal strain (LVLS) can be understood as longitudinal wall fractional shortening (LWFS) (in percentage), i.e. total MAPSE normalized by the LV length. We further proposed that LWFS correlates with and predicts LVLS.

## Methods

### Theoretical consideration

**Fig. 1:**
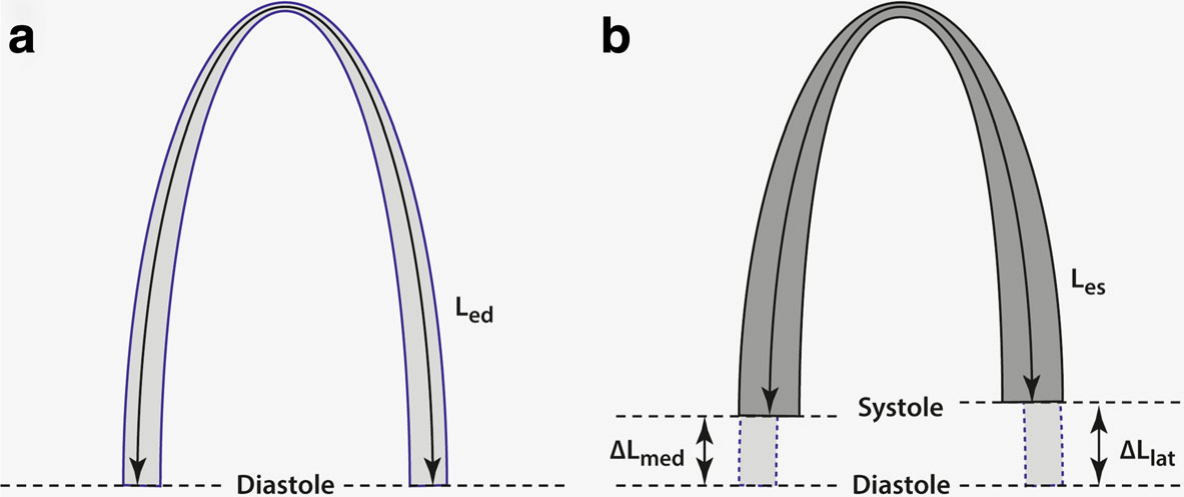
Definitions used in longitudinal strain calculation. Schematic diagram of the LV in A4C view. L_ed_ (**a**) and L_es_ (**b**) are the length of the entire U-shaped LV myocardium at end-diastole and end-systole. ΔL_med_ and ΔL_lat_ are the displacement of the medial (septal) and lateral mitral annuli, respectively

Echocardiographic strain is defined as the percentage change in length or width. From Fig. 1a, L_ed_ is the ventricular length for the entire U-shaped LV at end-diastole and L_es_ is the LV length at end-systole (Fig. 1). LVLS in the apical view is:

1 $$ LV\; longitudinal\kern0.17em strain\;(LVLS)=\frac{L_{es}-{L}_{ed}}{L_{ed}}\times 100\% $$

From Fig. 1b, (L_es_ – L_ed_) is the change in length, which is the sum of the change in length in both the medial (∆L_med_) and lateral (∆L_lat_) walls:

2 $$ {\displaystyle \begin{array}{c}{L}_{es}-{L}_{ed}=\left(-\Delta {L}_{med}\right)+\left(-\Delta {L}_{lat}\right)\\ {}{L}_{es}-{L}_{ed}=-\left(\Delta {L}_{med}+\Delta {L}_{lat}\right)\end{array}} $$

The negative sign represents myocardial shortening. LVLS can thus be re-written as:

3 $$ LVLS=-\frac{\left(\Delta {L}_{med}+\Delta {L}_{lat}\right)}{L_{ed}}\times 100\% $$

**Fig. 2:**
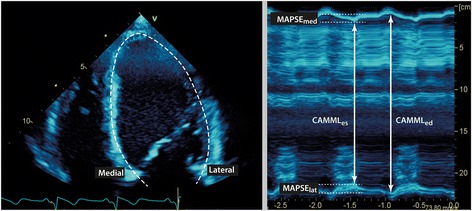
Curved anatomical M-mode (CAMM). A4C view of the LV at end-diastole (left). The dashed line represents the curved M-mode cursor along the middle of the myocardium at end-diastole. The corresponding CAMM of the entire U-shaped LV is shown on the right. The top of the CAMM represents the medial annulus (septum) and the bottom is the lateral annulus. *Abbreviations: CAMML*_*ed*_ and *CAMML*_*es*_ the LV length at end-diastole and end-systole, *MAPSE*_*med*_ and *MAPSE*_*lat*_ MAPSE of the medial and lateral annuli. *CAMML* curved-anatomical M-mode length, *MAPSE* mitral annular plane systolic excursion

**Fig. 3:**
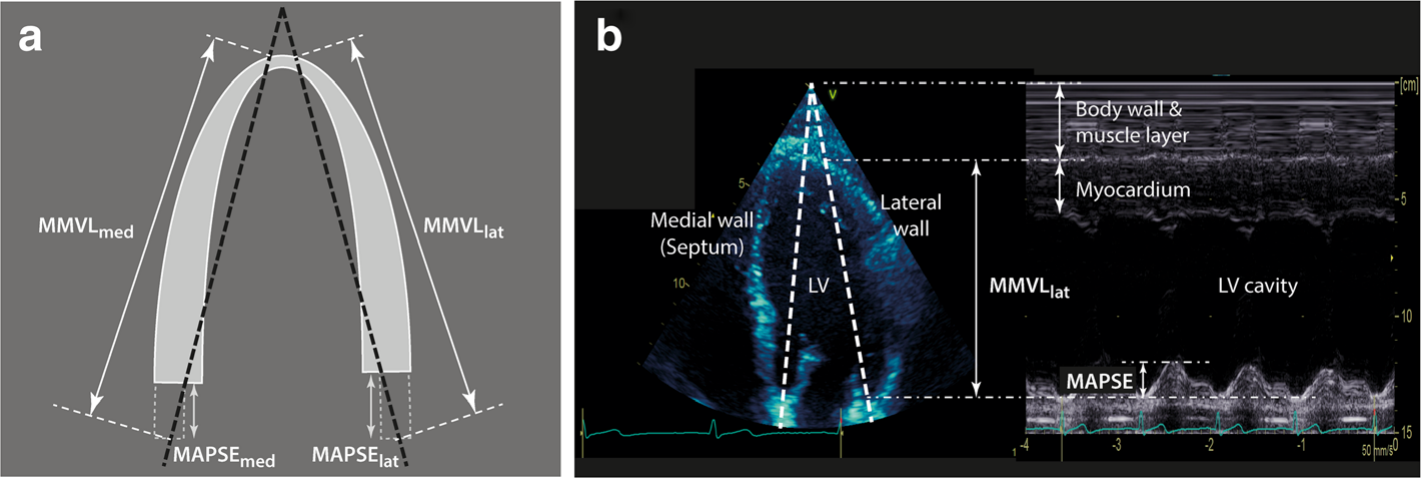
MAPSE and ventricular length obtained by conventional M-mode. **a** Schematic diagram of M-mode through the mitral annuli at end-diastole in the A4C view (white dashed line). M-mode ventricular length (MMVL) is the distance between the apex of the LV, where the cursor intersects the pericardium, to the mitral annulus at end-diastole. **b** An example of M-mode of the lateral wall in A4C view. Note the “static” horizontal lines at the top (superficial) layer are the body wall and muscle layer. *LV* left ventricle, *MAPSE* mitral annular plane systolic excursion, *MMVL* M-mode ventricular length

Theoretically, LVLS can be estimated by M-mode (motion-mode) measurements. Figures 2 and 3 show an example of curved anatomical M-mode (CAMM) and a conventional M-mode, respectively. CAMM, only available in some machines, collects M-mode information along a curved cursor. If traced along the LV myocardium in the apical longitudinal view, the motion of the whole U-shaped LV can thus be captured (Fig. 2). CAMML_ed_ and CAMML_es_ are the largest and smallest separations of the medial and lateral mitral annuli, and represent the end-diastolic length and end-systolic length of the LV, respectively (Fig. 2). We define CAMM fractional shortening (CAMMFS) as:

4 $$ CAMMFS=\frac{CAMM{L}_{ed}- CAMM{L}_{es}}{CAMM{L}_{ed}}\times 100\% $$

By comparing Eqs. (1) and (4), since CAMML_ed_ ≈ L_ed_ and CAMML_es_ ≈ L_es_, it follows that

5 $$ CAMMFS\approx - LVLS $$

MAPSE is the longitudinal excursion of the mitral annulus from end-diastole to end-systole (Fig. 3a). Comparing Figs. 1 and 3a, MAPSE_med_ and MAPSE_lat_ are approximately equal to ΔL_med_ and ΔL_lat_, respectively. Therefore,

6 $$ MAPS{E}_{sum}= MAPS{E}_{med}+ MAPS{E}_{lat}\approx \Delta {L}_{med}+\Delta {L}_{lat} $$

Figure 3b shows the actual images of an A4C view and conventional M-mode of the LV. MMVL is the end-diastolic ventricular length, after excluding the body wall and muscle layer, measured in M-mode. From Fig. 3a, the total M-mode left ventricular length (MMVL_total_) is:

7 $$ MMV{L}_{total}\approx MMV{L}_{med}+ MMV{L}_{lat} $$

LV longitudinal wall fractional shortening (LWFS) is defined as:

8 $$ LV\; LWFS=\frac{MAPS{E}_{sum}}{MMV{L}_{total}}\times 100\% $$

Since MMVL_total_ approximates L_ed_ (see Additional file 1), from Eqs. (3), (6) and (8),

9 $$ -\frac{\left(\Delta {L}_{med}+\Delta {L}_{lat}\right)}{L_{ed}}\approx \frac{MAPS{E}_{sum}}{MMV{L}_{total}} $$

That is,

10 $$ - LVLS\approx LWFS $$

### Study design and setting

This was a retrospective cross-sectional observational study with patients’ transthoracic echocardiography (TTE) records retrieved from the Intensive Care Unit Cardiovascular Ultrasound Laboratory in a tertiary hospital in Sydney.

### Study population

TTE studies between June to November 2016 were screened for suitability for inclusion into the study. To cover a wider range of LVLS, hence predictability, a priori decision was made to extend the range of LVLS by including approximately 40% of TTE studies that showed abnormal LV systolic function, defined as the presence of one or more segmental wall dysfunction or LV ejection fraction (LVEF) < 50%. Inclusion criteria were: (1) the TTE study must contain a apical 4-chamber (A4C) view with at least three cardiac cycles recorded, (2) the image quality must be of adequate quality to allow successful speckle tracking (low background noise and good delineation of endocardial border), (3) all LV inferoseptal and anterolateral segments and the mitral annulus must be visible throughout the cardiac cycle, (4) the LV long axis must lie along the midline of the sector for proper M-mode measurement, (5) the LV should not be foreshortened, (6) there should not be significant translational artefacts causing out-of-plane displacement, (7) the two-dimensional (B-mode) frame rate must be 50 fps or higher, and (8) the patient must be in sinus rhythm. A total of 127 studies (patients) were included in the first round of screening for LV systolic function. Of these, 65 patients had abnormal LV systolic function. Forty-seven studies were excluded upon further screening for quality. Most of these studies were excluded for more than one reason: low frame rate (*n* = 21), inadequate study quality (n = 31) and angulated heart axis (*n* = 15) were the main reasons. “Inadequate study quality” includes studies that did not satisfy any of points (2) to (7) of the inclusion criteria.

### Measurements

#### Transthoracic echocardiography

All included TTE studies were performed using GE Vivid 7 or E9 machine (GE Healthcare, Horton, Norway) and EchoPac software (version 201, Revision 61.0, GE Healthcare, Wauwatosa, WI, USA) was used for analysis. LVLS from the A4C were measured offline using speckle tracking. One complete cardiac cycle, excluding the first and the last cycles, was used in strain analysis. After optimizing the overall gain, the endocardial border was traced manually from the medial to the lateral mitral annulus making sure the trabeculae and papillary muscles were excluded. The width of the region of interest was adjusted to exclude the pericardium. The software automatically tracked the myocardial speckles and calculated the LVLS. For comparison, LVEF in this study was measured using Simpson’s monoplane method in the A4C view.

**Fig. 4:**
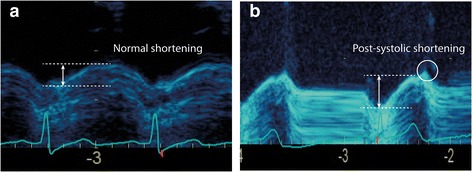
Post-systolic shortening in MAPSE. MAPSE without (**a**) and with (**b**) post-systolic shortening (circle)

CAMM was measured offline using the EchoPac software. The entire U-shaped LV was traced along the middle of the myocardium in the A4C view at end-diastole, including also the medial and lateral mitral annuli (Fig. 2). CAMML_ed_ and CAMML_es_ were the distances (ventricular lengths) between the medial and lateral annuli at end-diastole and end-systole, respectively. Inner edge was used in the measurements and post-systolic shortening was excluded when measuring CAMML_es_ [11] (Fig. 4).

MMVL and MAPSE of the medial and lateral walls were measured in the A4C view using the leading-edge method. End-diastolic MMVL was the M-mode distance between the apical pericardium to the mitral annulus (Fig. 3b). MAPSE was measured from the nadir (end-diastole) to the peak (end-systole) but avoiding post-systolic shortening (Fig. 4). MAPSE_sum_ and MMVL_total_ were calculated using Eqs. (6) and (7), respectively. If M-mode image was not available in the original study, post-processing M-mode was constructed from the A4C cineloop. M-mode measurements were performed separately to the LVLS measurements and the investigators (SJH and AMH) were blinded to the LVLS results at the time of measurements.

#### Statistics

The included studies (*n* = 80) were computer-randomized, by generating a set of randomized binary codes according to a uniform distribution, into a training set (*n* = 50) and a validation set (*n* = 30). Univariate generalized linear models were constructed from the training set using maximum likelihood estimation assuming Gaussian family distribution. LVLS was the response variable and MAPSE_sum_, CAMMFS or LWFS was the predictor:

$$ LVLS={b}_0+{b}_1(predctor)+\varepsilon $$

where b_0_ and b_1_ are the regression coefficients (the intercept and the slope, respectively) and ε represents random or measurement errors. Student *t* test was used to test if b_0_ and b_1_ were equal to zero. Model fitness was tested using Chi-square test and Hosmer-Lemeshow test. Model selection was also based on maximum likelihood pseudo-R^2^ (reported as R^2^), and dispersion (reported as mean squared error, MSE). Correlation between two variables was assessed using Pearson correlation. All models were diagnosed for linearity, residual normality and equal variance using QQ plot and residual versus fitted values plot to ensure model validity.

Predictive capability of the selected model was tested on the validation set by comparing the MSE from the training set with the mean squared prediction error (MSPE), which is defined as:

$$ MSPE=\frac{\sum {\left( LVL{S}_{meas}- LVL{S}_{pred}\right)}^2}{n} $$

where n is the sample size, and the subscripts *meas* and *pred* represent the measured (observed) and predicted values. Agreement between the LVLS_meas_ and LVLS_pred_ was analysed using the Bland and Altman method [12]. Intra- and inter-observer agreement and reliability were examined using Bland and Altman plot and intraclass correlation coefficient (ICC), respectively.

LVLS was presented as absolute (positive) values in this study. Measurement data were summarized as mean ± SD. LVLS and LWFS data were normally distributed for the normal and abnormal LV function groups (Shapiro-Wilk test, *P* > 0.05). Model parameters (such as intercepts and slopes) and test statistics were presented as mean ± SE or mean [upper, lower 95% confidence interval (CI)]. 95%CI was presented wherever possible and when effect size was more informative, otherwise *P* value was presented [13]. All analyses were carried out using the open source software R (version 3.3.1) (The R Foundation for Statistical Computing, Vienna, Austria).

#### Power and sample size

Sample size for the training cohort was estimated using a power (1 - β) of 0.90 and a critical significance level (α) of 0.005 to ensure reproducibility of the results [14, 15]. With one predictor and assuming a correlation (r) of 0.55 (R^2^ of 0.3) a sample size of 42 achieves a power of 0.90.

## Results

### Patient characteristics

A total of 80 patients’ records were included in this study and their characteristics are displayed in Table 1. Forty-seven patients (59%) were reported to have normal LV function with mean LVEF of 57 ± 5%. Thirty-three patients (41%) were reported to have LV dysfunction and the mean LVEF was 28 ± 11%. The patient characteristics for the training set and validation set are summarized in Table 2. The characteristics for the two data sets were similar.

**Table 1:** Patients characteristics

	All patients (*n* = 80)	Patients with normal LV systolic function (*n* = 47)	Patients with LV systolic dysfunction (*n* = 33)
Gender (M/F)	37/43	19/28	18/15
Age	61.5 ± 14.4	58.7 ± 12.7	65.6 ± 15.9
LVEF (%)	45 ± 16	57 ± 5	28 ± 11
LVEDV (ml)	107 ± 47	87 ± 31	136 ± 51
MAPSE_med_ (mm)	10.7 ± 4.5	13.2 ± 3.4	7.3 ± 3.6
MAPSE_lat_ (mm)	12.8 ± 4.4	15.0 ± 3.2	9.8 ± 4.1
Mean MAPSE (mm)	11.8 ± 4.3	14.1 ± 2.9	8.5 ± 3.7
LWFS (%)	12.3 ± 4.2	14.6 ± 2.5	9.1 ± 3.8
CAMMFS (%)	12.7 ± 4.5	15.3 ± 2.8	8.9 ± 4.7
LVLS (%)	-13.8 ± 5.4	-16.9 ± 3.4	-9.4 ± 4.7

*LVEF* LV ejection fraction, *LVEDV* LV end-diastolic volume, *MAPSE* mitral annular plane systolic excursion, *LWFS* longitudinal wall fractional shortening, *CAMMFS* curved-anatomical M-mode fractional shortening, *LVLS LV* longitudinal strain

**Table 2:** Patients characteristics for the training set and validation set

	Training set (*n* = 50)	Validation set (*n* = 30)
Gender (M/F)	23/27	14/16
Age	61.8 ± 14.1	61.1 ± 15.2
[min, max]	[21, 90]	[26, 91]
Medical conditions		
Congestive heart failure	3	3
Chronic kidney failure	1	0
Cardiogenic shock	1	0
Dilated cardiomyopathy	3	1
Ischemic heart disease	10	4
Pulmonary edema	0	1
Pulmonary embolism	1	0
Sepsis	0	1
Stroke	2	4
Takotsubo	1	0
Others	28	16
LVEF (%)	43 ± 18	49 ± 13
LVEDV (ml)	114 ± 51	95 ± 37
MAPSE_med_ (mm)	10.8 ± 4.9	10.7 ± 3.9
MAPSE_lat_ (mm)	12.7 ± 4.3	13.1 ± 4.6
Mean MAPSE (mm)	11.7 ± 4.4	11.9 ± 4.1
LWFS (%)	12.0 ± 4.5	12.9 ± 3.6
CAMMFS (%)	12.5 ± 4.8	12.8 ± 3.9
LVLS (%)	-13.4 ± 5.8	-14.5 ± 4.7

*LVEF* LV ejection fraction, *LVEDV* LV end-diastolic volume, *MAPSE* mitral annular plane systolic excursion, *LWFS* longitudinal wall fractional shortening, *CAMMFS* curved-anatomical M-mode fractional shortening, *LVLS LV* longitudinal strain

### Correlation matrix

**Fig. 5:**
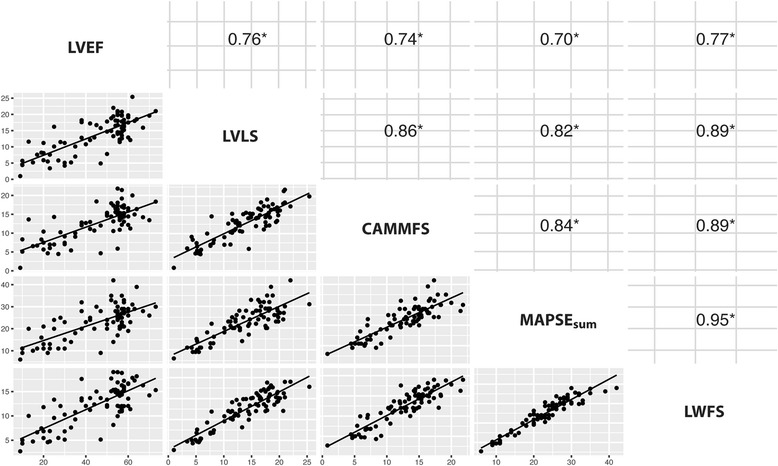
Correlation matrix of various variables. Correlation matrix between LV ejection fraction (LVEF), LV longitudinal strain (LVLS), CAMM fractional shortening (CAMMFS), sum of medial and lateral MAPSE (MAPSE_sum_), and longitudinal wall fractional shortening (LWFS) for all patients (n = 80). *P < 0.001. *CAMML* curved anatomical M-mode ventricular length, *LVEF* LV ejection fraction, *LVLS* LV longitudinal strain, *LWFS* longitudinal wall fractional shortening, *MAPSE* mitral annular plane systolic excursion

The correlation matrix between LV ejection fraction (LVEF), LVLS and various predictors for the whole data set (*n* = 80) is shown in Fig. 5. These variables showed good correlation with each other, although LVEF demonstrated the weakest correlations with the other measurements. LVLS shown good correlations with CAMMFS, MAPSE_sum_ and LWFS with *r* = 0.86, 0.82 and 0.89, respectively (*P* < 0.001 for all).

### Model building and selection from training set (*n* = 50)

**Fig. 6:**
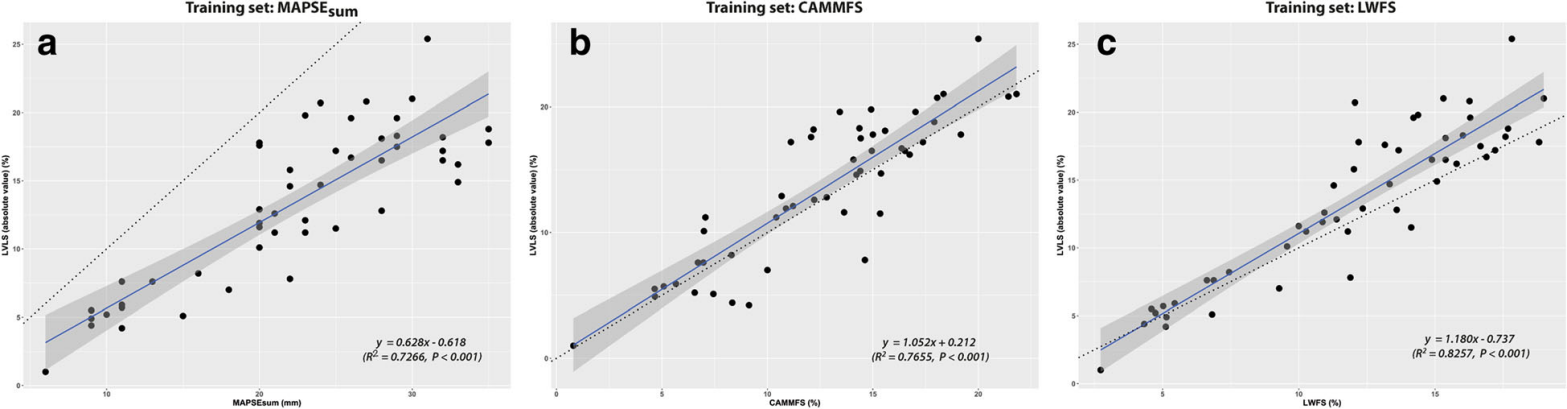
Correlations between LVLS and MAPSE_sum_, CAMMFS and LWFS for the training set. **a** MAPSE_sum_. **b** CAMMFS. **c** LWFS. The dotted line in each figure represents the line of equality, and the solid lines and shaded areas are the line of best fit ± 95%CI. *CAMML* curved anatomical M-mode ventricular length *LVLS* LV longitudinal strain, *MAPSE* mitral annular plane systolic excursion

Three models were built using MAPSE_sum_, CAMMFS or LWFS separately as predictor. The results are shown in Fig. 6a to c and Table 3. All three predictors showed good to very good correlations with LVLS with *r* = 0.852 [0.752, 0.914], 0.875 [0.780, 0.928] and 0.909 [0.844, 0.974] for MAPSE_sum_, CAMMFS and LWFS, respectively. The intercepts (b_o_) were not significantly different from zero and the slopes (b_1_) were greater than zero (Table 3).

**Table 3:** Models constructed from the training set (*n* = 50): parameters and statistics

Parameters and statistics	Model 1	Model 2	Model 3
Predictor	*MAPSE*_*sum*_	*CAMMFS*	*LWFS*
Intercept (b_o_)	-0.618 ± 1.315	0.212 ± 1.124	-0.737 ± 0.999
	(*P* = 0.640)	(*P* = 0.851)	(*P* = 0.822)
Slope (b_1_)	0.628 ± 0.056	1.052 ± 0.084	1.180 ± 0.078
	(*P* < 0.001)	(*P* < 0.001)	(*P* < 0.001)
R^2^	0.7266	0.7665	0.8257
MSE (dispersion)	9.34	7.98	5.95
GoF test* (*P* value)			
Δdeviance (χ^2^ test)	<0.001	<0.001	<0.001
HL test	>0.99	>0.99	>0.99

Intercepts and slopes are presented as mean ± SE. *Goodness-of-fit (GoF) tests. Δdeviance (χ^2^-test) compares the change in deviance from the null model (without predictor) to one containing the predictor. Smaller *P* value in indicates more significant change after adding the predictor. Hosmer-Lemeshow (HL) test examines the difference between the model and the observed data. Larger *P* value indicates no difference. *MAPSE* mitral annular plane systolic excursion, *CAMMFS* curved anatomical M-mode ventricular length, *LWFS* longitudinal wall fractional shortening, *SE* standard error, *MSE* mean squared error

While these models exhibited good to very good correlations between the predictor and LVLS, the data points for model 1 and 2 were more dispersed (variable) than model 3 as evident from the MSEs (9.34 vs 7.98 vs 5.95). LWFS (model 3) also explained the variability better than models 1 and 2 (i.e. largest R^2^) (Table 3). Model 3 was therefore used to predict LVLS in the validation set.

### Model validation using the validation set (*n* = 30)

The intercept (b_o_) and slope (b_1_) after fitting a regression line to the validation set were similar to model 3 (Tables 3 and 4). Although R^2^ was slightly lower than the training set (0.7305 vs 0.8257), the MSEs were similar and was reasonably small (6.10 vs 5.95).

**Table 4:** Relationship between LVLS and LWFS in the validation set (*n* = 30)

Parameters & statistics	Validation set
Predictor	*LWFS*
Intercept (b_o_)	0.169 ± 1.701
	(*P* = 0.922)
Slope (b_1_)	1.107 ± 0.127
	(*P* < 0.001)
R^2^	0.7305
MSE (dispersion)	6.10
GoF test* (*P* value)	
Δdeviance (χ^2^ test)	<0.001
HL test	>0.99

Intercepts and slopes are presented as mean ± SE. *LVLS* LV longitudinal strain, *LWFS* longitudinal wall fractional shortening, *SE* standard error, *MSE* mean squared error, *HL* Hosmer-Lemeshow

**Fig. 7:**
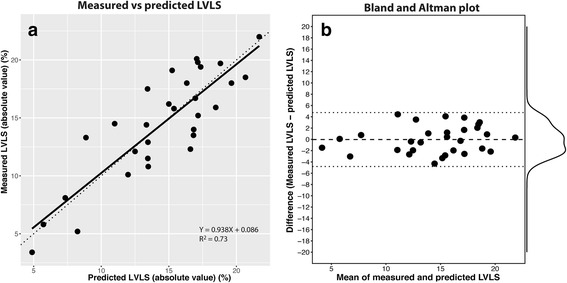
Prediction of LVLS by LWFS (model 3) in the validation set. **a** correlation between measured LVLS and predicted LVLS. **b** Bland and Altman plot of the difference of the measured and predicted values. The *long dashed line* is the mean of the differences and *dotted lines* are the limits of agreement. Marginal density plot of the difference is shown on the *right. LVLS* LV longitudinal strain

The mean LVLS_pred_ was 14.5 ± 4.3%, which was similar to LVLS_meas_ in the validation set (14.5 ± 4.7%). LVLS_pred_ exhibited a very good correlation with LVLS_meas_ (Fig. 7a). The slope = 0.938 [0.769, 1.107] and the intercept was not statistically significant from zero (0.860 [-1.59, 3.31]). MSPE was 5.75, which was similar to the MSE of the training set (model 3) indicating the absence of significant bias and had good prediction capacity.

Figure 7b shows the Bland and Altman plot for relationship between the difference and the mean between LVLS_meas_ and LVLS_pred_. The differences were normally distributed, and there was no bias in the prediction (mean difference = -0.03% [95%CI: -0.89, 0.95]). The 95% limits of agreement (LOA) were -4.75% and 4.82%.

### Intra- and inter-observer agreement and reliability of LWFS

The bias and LOA for intra-observer measurements of LWFS were 0.219 [-0.182, 0.621] and ± 3.539, respectively. The ICC was 0.91 [0.87, 0.94]. These indicate non-bias agreement and good intra-rater reliability. On the other hand, very small but insignificant bias was observed between two independent observers (bias = 0.589 [0.000, 1.176] and LOA = 3.196). Good inter-rater reliability was observed (ICC = 0.93 [0.91, 0.94]).

## Discussion

The present study shows that, using univariate linear model, both CAMMFS and LWFS exhibited very good correlations with LVLS in the apical 4-chamber view. Between CAMMFS and LWFS, the latter provides a better goodness-of-fit with LVLS. LWFS measurement was repeatable and reliable.

### Longitudinal strain

To date, strain studies in critically ill patients are scarce. Most studies were performed on intensive care septic patients [16]. The main consistent findings from these studies were that left ventricular longitudinal strain was more sensitive than LVEF in detecting systolic dysfunction, and longitudinal strain could not predict mortality in septic patients [3, 17–19]. The relatively small number of critical care studies available may reflect (1) the difficulties in obtaining optimal images for speckle tracking in this population, (2) speckle-tracking software is not available in the ultrasound machines, which are mostly used as a point of care device, and/or (3) most critical care clinicians are not trained in speckle-tracking strain measurements.

The present study supports the notion that LVLS and longitudinal M-mode indices (MAPSE, CAMMFS and LWFS) are closely related and highly correlated with each other. The results are not surprising because all of these indices, including LVLS, measure the motion of myocardial in the longitudinal plane. On the other hand, the correlation between LVEF and LVLS was also highly correlated but less ideal than M-mode indices (*r* = 0.76). A similar correlation between LVEF and LVLS (*r* = 0.7) has been reported recently [18]. One explanation for the poorer correlation is that LVEF reflect not only longitudinal contraction but also radial and circumferential contraction, whereas LVLS reflects mainly the longitudinal contraction.

### MAPSE

MAPSE was first described in 1932 by Hamilton and Rompf as caudal-cephalad movement of the atrioventricular plane [20]. Since the first ultrasound study in 1967, MAPSE has been reported as a consistent and reliable marker for longitudinal function of the LV [21]. MAPSE correlates with LVEF with reported r ranged from 0.55 to 0.95 [22–25]. The present study also found a good correlation between the MAPSE_sum_ with LVEF (*r* = 0.70). Although MAPSE only demonstrates the longitudinal motion of the LV, it was more sensitive than LVEF in detecting early LV dysfunction, such as in hypertensive patients [26, 27]. In patients with moderate to severe aortic stenosis, MAPSE was as good as GLS in detecting early LV dysfunction [28]. Similar to a previous report that showed a positive correlation between MAPSE and longitudinal strain [29], the present study also found a good correlation between MAPSE_sum_ with LVLS (*r* = 0.82).

### M-mode fractional shortening as longitudinal strain

Theoretically, LVLS, CAMMFS and LWFS measures the same phenomenon – the change in LV length normalized to its original (end-diastolic) length. In a study that purported to use MAPSE_lat_/left ventricular length (MAPSE/L) as an index for LV longitudinal function in children where adjusting for age-dependent ventricular length is important, GLS was found to be moderately correlated with MAPSE_lat_/L even when the study was not originally designed to investigate the relationship between the two (r = 0.56) [30].

The present study demonstrated that LWFS (MAPSE_sum_/MMVL_total_) exhibited very good correlation with LVLS in the training set (*r* = 0.909) providing supportive evidence that LWFS and LVLS are two closely related, if not similar, measurements. Using a separate validation set, LWFS displayed very good predictive capability (see Fig. 7). The 95% LOA of the difference between LVLS_meas_ and LVLS_pred_ was -0.03 ± 4.78%, which was better than the variability (SD) of longitudinal strain (see Tables 1 and 2) and was within the test-retest limit reported for longitudinal strain (2.5% to 5.0%) [31]. Of note, the value of LWFS should not be taken the same as LVLS. In calculating LWFS, we assumed that MMVL_total_, which was measured in a straight line, was the same as the curved ventricular length (L_ed_) used in LVLS measurement. Theoretically, MMVL_total_ is always less than L_ed_. However, in an average size LV, MMVL_ed_ and the L_ed_ differ by less than 10% (see Additional file 1).

### Strengths and weaknesses LWFS compared to strain

Compared to strain measurement, LWFS is less dependent on image quality. This is especially important in difficult patients such as obese or critically ill patients. However, M-mode is angle-dependent, therefore, a good alignment of the LV axis with the midline of the sector is necessary for accurate measurement. LWFS does not give segmental information. Yet, there are other advantages of LWFS measurement: it can be performed quickly even on the bedside, requires minimal training, and can be averaged over several consecutive cardiac cycles, which is very useful in irregular rhythm. M-mode has a very high sampling rate, which is typically between 1000 to 2000 samples per second, and provides superior temporal resolution [10]. Good intra- and inter-rater agreement and reliability makes follow-up and cross-platform M-mode studies comparable. Special software is not required for LWFS measurements and can be performed using any point-of-care machines.

On the other hand, strain relies on optimal image quality which is often not obtainable from every patient or view. Speckle tracking also relies on high frame rate, while slow frame rate or high heart rate may limit tracking accuracy. Sampling rate is not an issue with M-mode (see above). Unlike M-mode, averaging over consecutive cardiac cycles is time-consuming and impossible in irregular cardiac rhythm. However, speckle-tracking strain measurement is less susceptible to angle and translational artefacts and also gives segmental information. That said, false positives of segmental wall information have been described [32]. Inter-vendor differences in speckle-tracking algorithm is also a major concern [31]. Finally, special costly software is usually required for speckle tracking.

### Clinical perspective

This study implies that LWFS measurement offers an alternative measurement or method of estimating LVLS. As LWFS measures the longitudinal motion of the left ventricle, in theory, it may act as a prognostic tool and offers similar sensitivity in detecting early LV systolic dysfunction as LVLS. It has the potential to be used as a follow-up tool for subclinical myocardial dysfunction and to evaluate the treatment effects. In this regard, LWFS can be useful in deciding when to initiate or terminate inotropes.

### Limitations of the study

Inter-vendor inconsistencies are known to be a major issue in strain measurements and may affect the applicability of the prediction model (equation). The inconsistencies are mainly due to different definitions and algorithms used by different vendors [9, 31]. Even with the same vendor, different versions of speckle-tracking software have been shown to yield different GLS values [9]. Therefore, the prediction model used in this study may not be applicable to different system or versions of software. However, when used as index itself, LWFS does not suffer from such problems.

As this was a retrospective study, we were unable to determine the true feasibility of measuring LWFS in ICU patients. Many echocardiograms (37%) were not optimized for the purpose of measuring LWFS and LVLS in this study. As a result, they were excluded due to inadequate image optimization, low frame rate, deviated heart axis and foreshortened apical view. Although patient characteristics played a contributory role in image quality, the experience of the echocardiographers, some of whom were receiving basic level critical care echo training, also contributed. We expect the feasibility of measuring LWFS should improve with experience. Also, as the apical 2 and 3 chamber views were not optimized for strain or M-mode measurement purposes, we were not able to obtain GLS and a “global” LWFS value for comparison. Theoretically, “global” LWFS should have a similar predictability capacity as LWFS in this study. Unfortunately, we were unable to determine the true feasibility of LWFS measurements when compared to LVLS, which is best done in a prospective study on consecutive patients. We were also unable to track the changes in LV systolic function with treatment.

For the purpose of this association study, the patients (studies) included in this study were not randomly selected and hence subject to selection bias. First, we selected only those studies which were optimal for LVLS and M-mode measurements, hence might have excluded those very sick and difficult patients. Second, to extend the predictable range, we deliberately included a large proportion of patients with abnormal LVEF (approximately 40%), thereby creating two distinct populations. These patients were unlikely to represent the usual mix of ICU patients. We omitted presenting the clinical data and treatment data which could be biased and misleading in this study. Of note, this selection bias did not affect the validity of the model as diagnostic tests on the assumptions of normality, equal variance and linearity were not violated.

## Conclusions

This study demonstrated that LWFS is an unbiased predictor of LVLS. In fact, indices that measured LV longitudinal function, namely MAPSE, CAMMFS and LWFS, displayed good correlations with longitudinal strain in this study. Compared to speckle-tracking strain measurements, LWFS only requires simple M-mode measurements which are reproducible and reliable, requires minimal training and are available in all machines. LWFS could potentially be a useful index for clinical use. Research into the clinical utility of LWFS is however required.

## Additional file

Additional file 1: The difference between the M-mode ventricular length (MMVL) and LV length based on a semi-elliptical model. Theoretical (mathematical) consideration of the difference of ventricular length between conventional M-mode and a semi-elliptical model. (PDF 2953 kb)

## Abbreviations

CAMMFS: Curved-anatomical M-mode fractional shortening
CAMML: Curved anatomical M-mode ventricular length
GLS: Global longitudinal strain
ICC: Intraclass correlation coefficient
LOA: Limits of agreement
LV: Left ventricle
LVEDV: LV end-diastolic volume
LVEF: LV ejection fraction
LVLS: LV longitudinal strain
LWFS: Longitudinal wall fractional shortening
MAPSE: Mitral annular plane systolic excursion
MMVL: M-mode ventricular length
MSE: Mean squared error
MSPE: Mean squared prediction error
