# Supplementary material for: Longitudinal wall fractional shortening: an M-mode index based on mitral annular plane systolic excursion (MAPSE) that correlates and predicts left ventricular longitudinal strain (LVLS) in intensive care patients
Source: Crit Care. 2017 Nov 25;21:292. doi: 10.1186/s13054-017-1876-x (PMC5702151; doi:10.1186/s13054-017-1876-x)
Supplement: Additional file 1: — The difference between the M-mode ventricular length (MMVL) and LV length based on a semi-elliptical model. Theoretical (mathematical) consideration of the difference of ventricular length between conventional M-mode and a semi-elliptical model. (PDF 2953 kb) [file 13054_2017_1876_MOESM1_ESM.pdf]

Additional file 1

The difference between the M-mode ventricular length (MMVL) and LV length based on a semi-elliptical model

Approximating the end-diastolic left ventricle (LV) length

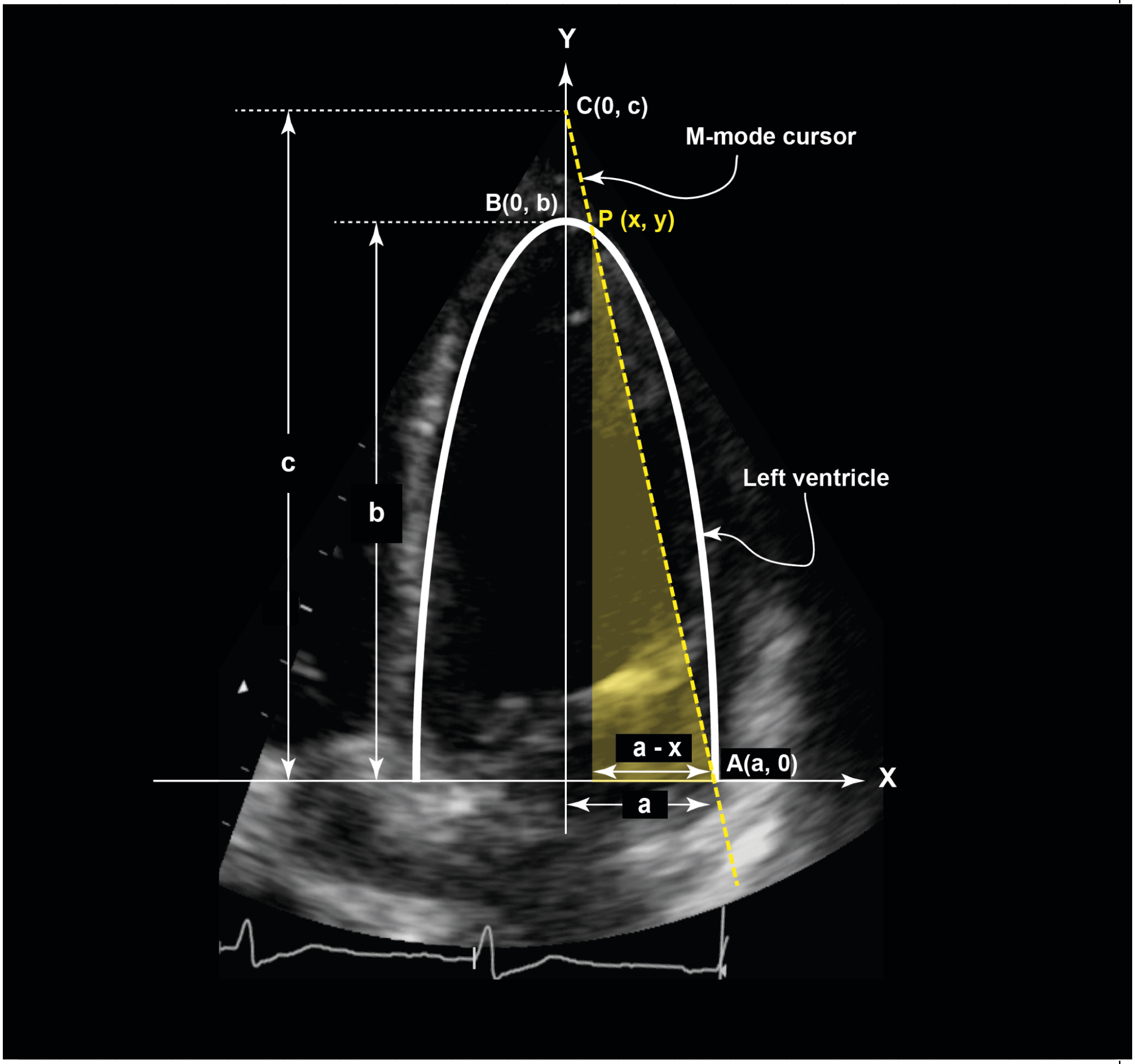

FIGURE S 1: MODELLING OF LV BY A SEMI-ELLIPSE

A semi-ellipse (thick white line) with X-Y co-ordinate is superimposed on an A4C 2D image of the LV at end-diastole. The thick white line represents the LV and yellow dashed line represents the M-mode cursor. The cursor intersects the LV at point P (x, y) and the X-axis at point A (a, 0) which is the lateral annulus. The length of the LV is b, the radius is a, and the depth of the cursor to the mitral valve plane is c. MMVL is the length between point P (x, y) and A (a, 0).

If the LV at end-diastole is assumed to follow a semi-elliptical shape (**Figure S1**, thick white line), with

major axis radius (LV height) = b

minor axis radius (LV radius) = a

then the length (circumference) of the LV at end-diastole (L_{ed}) can be approximated by:

$$L_{ed} = \frac{\pi(a+b)}{2}\left(1 + \frac{u^2}{4} + \frac{u^4}{64} + \frac{u^6}{256} + \frac{25u^8}{16384}\right)$$

where

$$u = \frac{(a-b)}{(a+b)}$$

Finding the M-mode lateral ventricular length at end-diastole ($MMVL_{lat}$)

Referring to **Figure S1** where the yellow dashed line is the M-mode cursor, which starts at point C(0, c) and intersects the LV near the apex at point P(x, y) and ends at the mitral annulus at point A(a, 0) (X-intercept). The co-ordinate x and y can be found by solving the following equations:

$$\begin{cases} \frac{x^2}{a^2} + \frac{y^2}{b^2} = 1 \cdots\cdots\cdots\cdots\cdots\cdots\cdots\cdots\cdots\cdots\cdots (1) \\ y = -\left(\frac{c}{a}\right)x + c \cdots\cdots\cdots\cdots\cdots\cdots\cdots\cdots\cdots\cdots (2) \end{cases}$$

where c is the depth of the mitral annulus from transducer. Equations (1) and (2) are the equations for ellipse and straight line, respectively. After solving (1) and (2), the solutions for (x, y) are:

$$\begin{cases} x = \frac{(ac^2 - ab^2)}{(b^2 + c^2)} \cdots\cdots\cdots\cdots\cdots\cdots\cdots\cdots\cdots (3) \\ y = \frac{2b^2c}{(b^2 + c^2)} \cdots\cdots\cdots\cdots\cdots\cdots\cdots\cdots\cdots\cdots (4) \end{cases}$$

From **Figure S1**, using Pythagoras Theorem, $MMVL_{lat}$ is:

$$MMVL_{lat} = \sqrt{(a-x)^2 + y^2}$$

By substitution (3) and (4) into the above and after simplifying:

$$MMVL_{lat} = \frac{2b^2\sqrt{a^2 + c^2}}{(b^2 + c^2)}$$

Assuming the M-mode ventricular length for the medial (septal) wall is the same as the $MMVL_{lat}$, then

$$MMVL_{sum} = 2 \times \left(\frac{2b^2\sqrt{a^2 + c^2}}{(b^2 + c^2)}\right)$$

It can be seen that $MMVL_{sum}$ is affected by ventricular height (b), ventricular radius (a) and the depth of the mitral annulus from the transducer (c).

Underestimation by $MMVL_{sum}$

Underestimation of ventricular length by M-mode (Difference) can be calculated by:

$$Difference = L_{ed} - MMVL_{sum}$$

and the percentage difference of underestimation is:

$$Difference\% = \frac{(L_{ed} - MMVL_{sum})}{L_{ed}} \times 100\%$$

Figure S2 shows the percentage underestimation of ventricular length by M-mode. The figures demonstrated that (1) the longer the LV the smaller the underestimation, and (2) larger diameter results in larger underestimation.

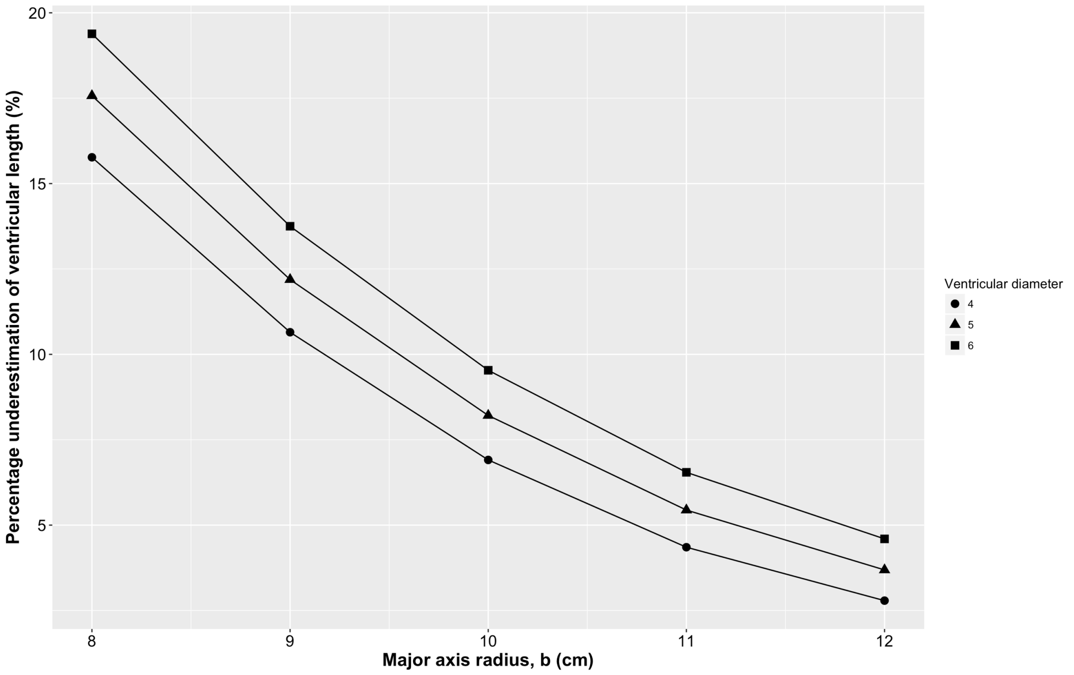

FIGURE S 2 PERCENTAGE UNDERESTIMATION OF VENTRICULAR LENGTH BY M-MODE
The percentage underestimation decreases with LV length but increases with LV diameter.

For an average adult size ventricle where the length (major axis diameter) is about 10 cm and a diameter of about 5 cm, the percentage underestimation is about 8%.
